# Gray Matter Deterioration Pattern During Alzheimer's Disease Progression: A Regions-of-Interest Based Surface Morphometry Study

**DOI:** 10.3389/fnagi.2021.593898

**Published:** 2021-02-03

**Authors:** Zhanxiong Wu, Yun Peng, Ming Hong, Yingchun Zhang

**Affiliations:** ^1^School of Electronic Information, Hangzhou Dianzi University, Hangzhou, China; ^2^Department of Biomedical Engineering, University of Houston, Houston, TX, United States

**Keywords:** gray matter, surface morphometry, Alzheimer's disease, cognition impairment, magnetic resonance imaging

## Abstract

Accurate detection of the regions of Alzheimer's disease (AD) lesions is critical for early intervention to effectively slow down the progression of the disease. Although gray matter volumetric abnormalities are commonly detected in patients with mild cognition impairment (MCI) and patients with AD, the gray matter surface-based deterioration pattern associated with the progression of the disease from MCI to AD stages is largely unknown. To identify group differences in gray matter surface morphometry, including cortical thickness, the gyrification index (GI), and the sulcus depth, 80 subjects from the Alzheimer's Disease Neuroimaging Initiative (ADNI) database were split into healthy controls (HCs; *N* = 20), early MCIs (EMCI; *N* = 20), late MCIs (LMCI; *N* = 20), and ADs (*N* = 20). Regions-of-interest (ROI)-based surface morphometry was subsequently studied and compared across the four stage groups to characterize the gray matter deterioration during AD progression. Co-alteration patterns (Spearman's correlation coefficient) across the whole brain were also examined. Results showed that patients with MCI and AD exhibited a significant reduction in cortical thickness (*p* < 0.001) mainly in the cingulate region (four subregions) and in the temporal (thirteen subregions), parietal (five subregions), and frontal (six subregions) lobes compared to HCs. The sulcus depth of the eight temporal, four frontal, four occipital, and eight parietal subregions were also significantly affected (*p* < 0.001) by the progression of AD. The GI was shown to be insensitive to AD progression (only three subregions were detected with a significant difference, *p* < 0.001). Moreover, Spearman's correlation analysis confirmed that the co-alteration pattern of the cortical thickness and sulcus depth indices is predominant during AD progression. The findings highlight the relevance between gray matter surface morphometry and the stages of AD, laying the foundation for *in vivo* tracking of AD progression. The co-alteration pattern of surface-based morphometry would improve the researchers' knowledge of the underlying pathologic mechanisms in AD.

## Introduction

Alzheimer's disease (AD) is a neurodegenerative disorder and the most common cause of dementia, which presumably starts with the aggregation of amyloid beta (Dicks et al., 2019). Gray matter volume reductions, a prominent AD feature because of neuronal loss, are considered as a close biological substrate of decline in cognitive functions. The decreases in gray matter volume can be measured by MRI. Studies have indicated gray matter abnormalities in patients with AD (Karas et al., 2004). Compared with healthy controls (HCs), patients with AD showed significantly lower global gray matter volume, lower whole brain volume, and greater ventricles (Guo et al., 2010). As the disease advances, gray matter abnormalities start to spread from the bilateral hippocampus, the amygdala, the entorhinal cortex, the posterior cingulate gyrus, and the medial thalamus to the parietal and frontal lobes (Moller et al., 2013). The symptomatic pre-dementia stage of AD, most commonly referred to as mild cognitive impairment (MCI), is critical to the development of predictive methods for early detection of AD and for further intervention programs (Li et al., 2018; Li K. et al., 2019; Ottoy et al., 2019; Wee et al., 2019). Different machine learning methods have been proposed to discriminate MCIs from HCs and ADs, based on the features extracted from structural MRI (Dimitriadis et al., 2017; Gomez-Sancho et al., 2018; Hojjati et al., 2018; Liu et al., 2020). Furthermore, recent evidence (Dicks et al., 2018; Tijms et al., 2018; Li R. et al., 2019; Wang et al., 2020) suggest that neuronal alterations in brain disorders tend to form patterns that resemble those of cerebral connectivity (co-alteration patterns). Therefore, to monitor disease progression, powerful non-invasive biomarkers, such as gray matter diffusivity (Jacobs et al., 2013) and gray matter volume (Lee et al., 2016; Qian et al., 2019), as well as their co-alteration patterns across the whole brain, are necessary to identify AD at early MCI stage and to advance the diagnosis, treatment, and prevention of these disorders.

Voxel-based morphometry (VBM) has been frequently used to examine gray matter differences across the whole brain. Using VBM, gray and white matter volume reductions were simultaneously detected between HCs and ADs (Baxter et al., 2006; Guo et al., 2010; Ha et al., 2012; Beejesh et al., 2019). An AD progression model was proposed to provide anatomically specific predictions of disease spread over time with VBM (Phillips et al., 2018). Dicks et al. (2019) modeled the gray matter atrophy in AD as a function of time and aging using Mini-Mental State Examination (MMSE) and found that the association of atrophy with MMSE was weaker than those with time or age. Based on VBM, local gray matter volumes were compared between patients with late- and early-onset AD and older and younger control subjects (Moller et al., 2013, Wu et al., 2020), and interactions of age and diagnosis on the volumes of the hippocampus and the precuneus were assessed, suggesting that the patterns of atrophy might vary in the spectrum of AD (Moller et al., 2013). Besides gray matter volume, revealing cross effects between AD-related incipient lesions helps to understand the progression to AD from MCI. Machine learning models were trained on VBM and connectome estimates to detect accurately AD-related neurodegeneration across the whole brain in a data-driven manner (Wang et al., 2019). Association between regional gray matter volume and two subtypes of psychotic symptoms in patients with mild AD was investigated, showing a distinct neural correlation between the paranoid and the non-psychosis groups (Lee et al., 2016). With the VBM technique, Cauda et al. (2018) found that structural alterations in the gray matter tended to follow the network-like patterns, indicating that structural co-alterations were influenced by connectivity constraints rather than being randomly distributed. Manuello et al. (2017) have investigated gray matter co-alterations of AD and found a series of co-altered areas that include the left hippocampus, left and right amygdalae, right parahippocampal gyrus, and right temporal inferior gyrus. Based on VBM, these studies consistently showed a widespread gray matter co-atrophy pattern due to AD. The co-alteration pattern may accelerate the development of neuronal abnormalities.

Unlike VBM, the surface-based morphometry methods can measure the cortical thickness and folding patterns, as well as the shape or curvature measures derived from brain surface meshes (Gutman et al., 2009; Lui et al., 2010). Previous studies demonstrated an increased accuracy of brain registration using brain surface meshes for spatial registration, compared to volume-based registration (Desai et al., 2005). Brain surface meshes permit new forms of analyses, such as the GI and the sulcus index, which measure surface complexity in 3D (Yotter et al., 2011) or cortical thickness (Righart et al., 2017). In addition, inflation or spherical mapping of the cortical surface mesh raises the buried sulci to the surface so that the mapped functional activity in these regions can be made visible. However, few studies have attempted to monitor gray matter alterations associated with MCIs and ADs using regions-of-interest (ROI)-based surface morphometry based on brain surface meshing. In this study, we investigated ROI-based surface morphometry of gray matter in different stages of AD, including HC, EMCI, LMCI, and AD, aiming to identify characteristic gray matter alteration patterns in terms of cortical thickness, GI, and the sulcus depth during AD progression.

## Methods and Materials

### Subjects

**Figure 1:**
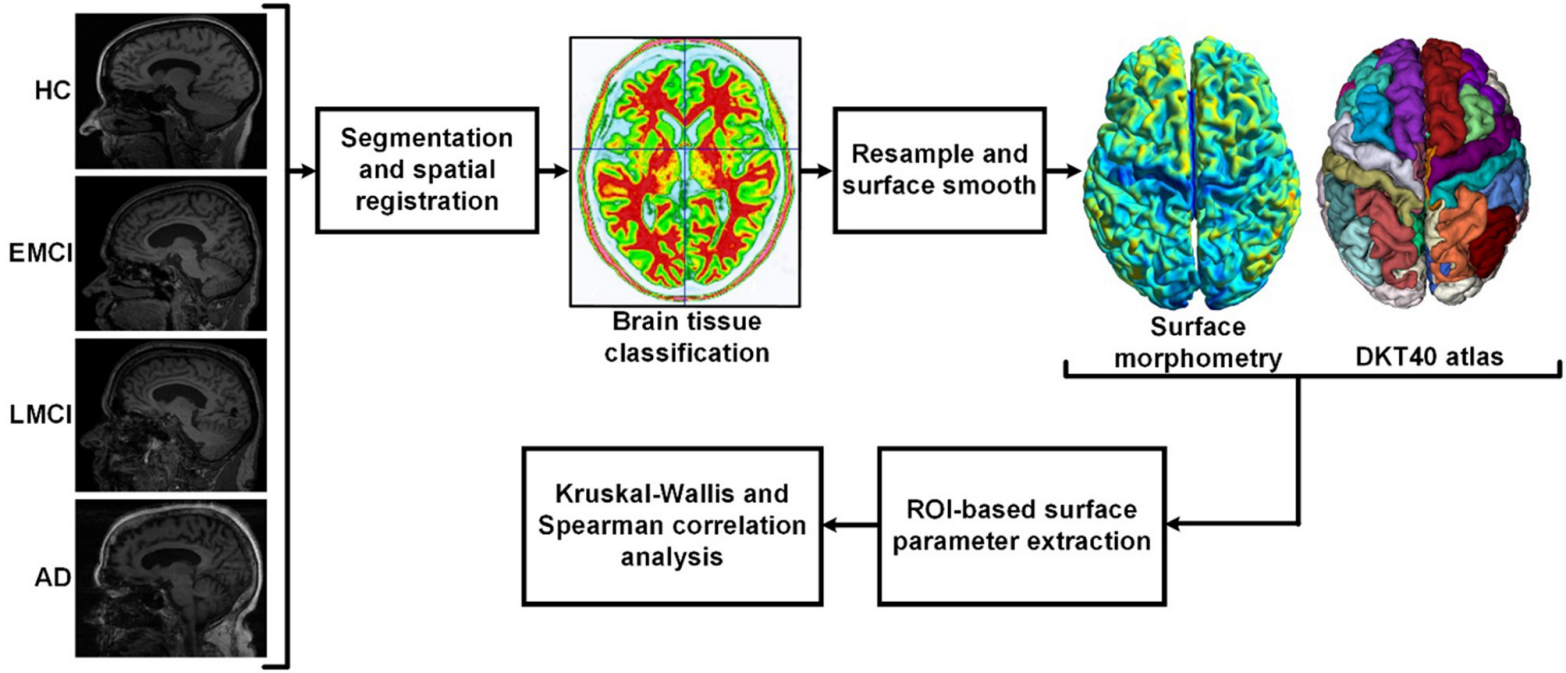
Flowchart of the regions-of-interest (ROI)-based surface morphometry analysis. After brain extraction and segmentation (white matter, gray matter, and cerebrospinal fluid), spatial normalization was performed to correct the orientation and the size of the brain. Then, the surface of gray matter was resampled and smoothed. The ROI-based surface parameters were extracted according to the DKT40 parcellation atlas. Cortical thickness, gyrification index (GI), and sulcus depth were used to characterize the deterioration patterns of gray matter during Alzheimer's disease (AD) progression.

Data used in this study were obtained from the Alzheimer's Disease Neuroimaging Initiative (ADNI) database (http://adni.loni.usc.edu). The ADNI was initially launched in 2004. The primary goal of ADNI is to identify MRI, PET, biomarkers, and genetic characteristics that would support the early detection and tracking of AD and improve the clinical trial design (Risacher et al., 2009; Jack et al., 2010a; Petersen et al., 2010). Scans were acquired with a 3.0-T head-only Siemens Medical Solutions MRI scanner (Erlangen, Germany). T1-weighted imaging parameters were: repetition time = 2,250 ms, echo time = 2.6 ms, flip angle = 9, field of view = 256 × 256 mm, acquisition matrix = 256 × 256, voxel size = 1 mm isotropic, and number of slices = 192. The demographic data of the subjects are summarized in Table 1. The flowchart of the ROI-based surface morphometry analysis is shown in Figure 1.

**Table 1:** Demographics of healthy controls (HCs), mild cognition impairments (MCIs), and Alzheimer's disease (ADs).

	**HC**	**Early MCIs (EMCI)**	**Late MCIs (LMCI)**	**AD**
Number of subjects	20	20	20	20
Gender	12F:8M	8F:12M	9F:11M	11F:9M
Mean age (Std)	73.75 (4.78)	75.95 (7.12)	74.35 (5.72)	74.85 (8.27)
CDR	0	0.5	0.5	0.5–1
MMSE	24–30	24–30	24–30	20–26

### Regions-of-Interest-Based Surface Morphometry

**Figure 2:**
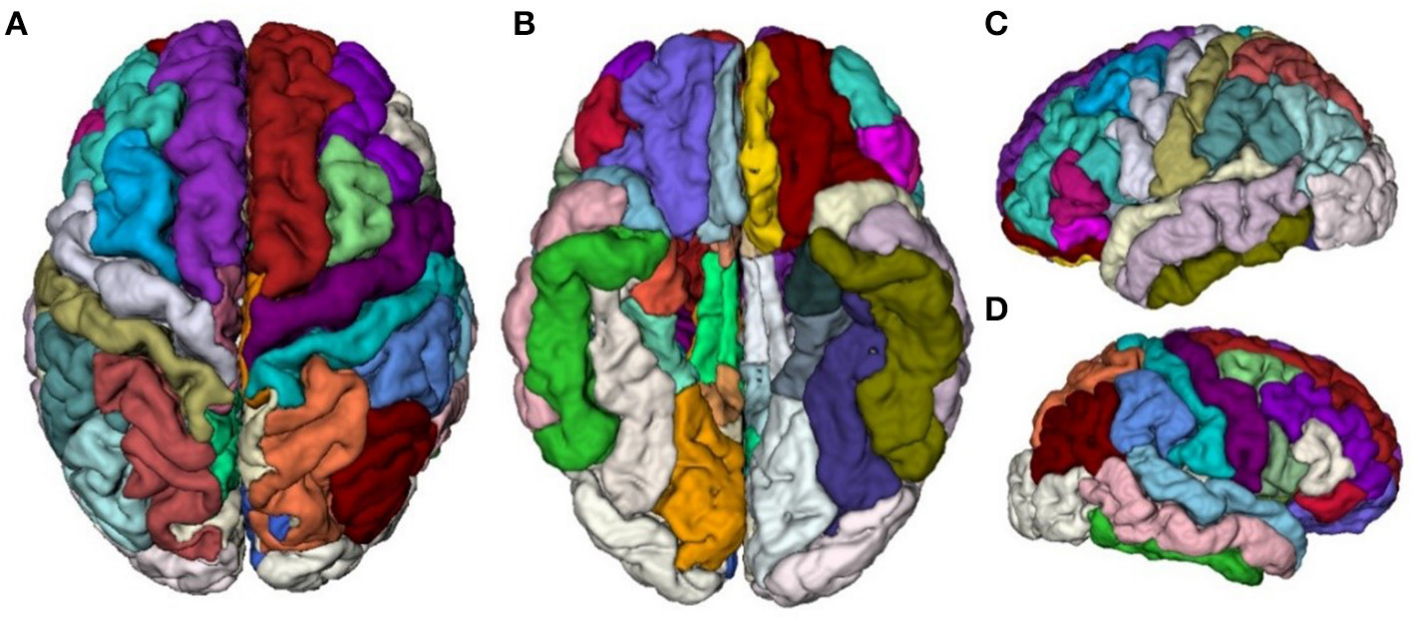
Visualization of the DKT40 cortical parcellation atlas, comprising 68 local regions. **(A)** Top view, **(B)** Bottom view, **(C)** Right view, and **(D)** Left view.

T1-weighted MR image preprocessing was performed using automated procedures included in the Computational Anatomical Toolbox (CAT12), an extension to the Statistical Parametric Mapping (SPM12) package (http://www.neuro.uni-jena.de/cat/). First, T1-weighted images were preprocessed with intensity normalization and skull stripping, followed by the normalization of the head position along the commissural axis and the labeling of the cortical and subcortical regions. Second, the images were segmented into gray matter, white matter, and cerebrospinal fluid with the parameter of Markov random fields set to 2, and co-registered to a probabilistic brain atlas with non-linear morphing. According to the probability that a given location is of a particular tissue class (gray matter, white matter, and cerebrospinal fluid), the intensity of the image at the location, and the local spatial configuration of the location related to the labels, each MRI voxel was assigned to one specific tissue class (Dahnke et al., 2013). In this process, all T1-weighted images were spatially normalized using combinations of affine linear transformation and non-linear registration to the standard Montreal Neurological Institute (MNI) template and segmented into gray matter, white matter, and cerebrospinal fluid. Third, a DKT40 labeling atlas was warped from standard space to subject space using the subject-specific inversed normalization parameters. All results were estimated in the native space before spatial normalization. Last, an individual brain atlas that consisted of 68 different gray matter areas was created for each participant according to the DKT40 parcellation atlas, as shown in Figure 2. The names and the corresponding indices of the parcellated regions are reported in Table 2. The pipeline used topology correction and spherical mapping to handle the partial volume effect, sulcal blurring, and asymmetry (Righart et al., 2017).

**Table 2:** Names and indices of the DKT40 parcellated cortical regions.

**Region**	**Region**	**Region**	**Region**
1 bankssts_left	2 bankssts_right	3 caudalanteriorcingulate_left	4 caudalanteriorcingulate_right
5 caudalmiddlefrontal_left	6 caudalmiddlefrontal_right	7 cuneus_left	8 cuneus_right
9 entorhinal_left	10 entorhinal_right	11 fusiform_left	12 fusiform_right
13 inferiorparietal_left	14 inferiorparietal_right	15 inferiortemporal_left	16 inferiortemporal_right
17 isthmuscingulate_left	18 isthmuscingulate_right	19 lateraloccipital_left	20 lateraloccipital_right
21 lateralorbitofrontal_left	22 lateralorbitofrontal_right	23 lingual_left	24 lingual_right
25 medialorbitofrontal_left	26 medialorbitofrontal_right	27 middletemporal_left	28 middletemporal_right
29 parahippocampal_left	30 parahippocampal_right	31 paracentral_left	32 paracentral_right
33 parsopercularis_left	34 parsopercularis_right	35 parsorbitalis_left	36 parsorbitalis_right
37 parstriangularis_left	38 parstriangularis_right	39 pericalcarine_left	40 pericalcarine_right
41 postcentral_left	42 postcentral_right	43 posteriorcingulate_left	44 posteriorcingulate_right
45 precentral_left	46 precentral_right	47 precuneus_left	48 precuneus_*right*
49 rostralanteriorcingulate_left	50 rostralanteriorcingulate_right	51 rostralmiddlefrontal_left	52 rostralmiddlefrontal_right
53 superiorfrontal_left	54 superiorfrontal_right	55 superiorparietal_left	56 superiorparietal_right
57 superiortemporal_left	58 superiortemporal_right	59 supramarginal_left	60 supramarginal_right
61 frontalpole_left	62 frontalpole_right	63 temporalpole_left	64 temporalpole_right
65 transversetemporal_left	66 transversetemporal_right	67 insula_left	68 insula_right

In this study, three ROI-based surface morphometry parameters were used to characterize the deterioration pattern of gray matter, including cortical thickness, the GI, and the sulcus depth:

#### Cortical Thickness

It is defined as the distance between the inner and the outer surface estimated from brain surface meshes, was related to cortical development (Dahnke et al., 2013), and identified as an important biomarker for normal development and aging (Sowell et al., 2004, 2007; Fjell et al., 2006) and pathological changes such as AD (Kuperberg et al., 2003; Sailer et al., 2003; Thompson et al., 2004; Rosas et al., 2008). Here, brain tissue segmentation was used to estimate the white matter distance and to project the local maxima (which is equal to the cortical thickness) onto other gray matter voxels using a neighboring relationship described by the white matter distance. This projection-based thickness allowed the handling of partial volume information, sulcal blurring, and sulcal asymmetries without explicit sulcus reconstruction (Dahnke et al., 2013).

#### Gyrification Index

It is defined as the ratio of the inner surface size to the outer surface size of an outer (usually convex) hull and was computed by averaging the absolute curvature values from each vertex of the spherical surface mesh (Luders et al., 2006).

#### Sulcus Depth

It is extracted based on the Euclidean distance between the central surface and its convex hull. Transformation with square root is used to render the data more normally distributed.

These surface parameters were estimated using the CAT toolbox (designed by Structural Brain Mapping Group, Departments of Psychiatry and Neurology, Jena University Hospital, Germany), which uses an internal interpolation to provide more reliable results even with low-resolution images and anisotropic spatial resolutions. Although interpolation cannot add more details to the images, the computations would benefit from the higher number of voxels, and the strip artifacts in preprocessed images are greatly reduced. While cortical thickness was estimated from the surface smoothed to 15 mm of full width at half maximum Gaussian kernel, GI and sulcus depth were computed from the surface smoothed with 20 mm full width at half maximum (Dahnke et al., 2013).

### Statistical Analysis

For each region (total 68 regions in DKT40 atlas), the gender covariate was first regressed out. Group-wise differences in cortical thickness, the GI, and the sulcus depth were assessed using the Kruskal–Wallis test. To additionally characterize the structural co-alterations in the evolution of AD, the Spearman's correlation analysis was used to investigate whether the alteration of a brain area was associated with the alteration of other brain areas. Statistical analyses were performed in MATLAB. For all analyses, significance was set at the value of *p* < 0.001 (uncorrected). Effect sizes for the Kruskal–Wallis tests can be defined as the chi-squared statistic divided by (*N* − 1).

(1) $$\begin{array}{l} \eta^{2}=\frac{\chi^{2}}{N-1} \end{array}$$

where χ^2^ is chi-squared statistic and *N* is sample size.

## Results

**Figure 3:**
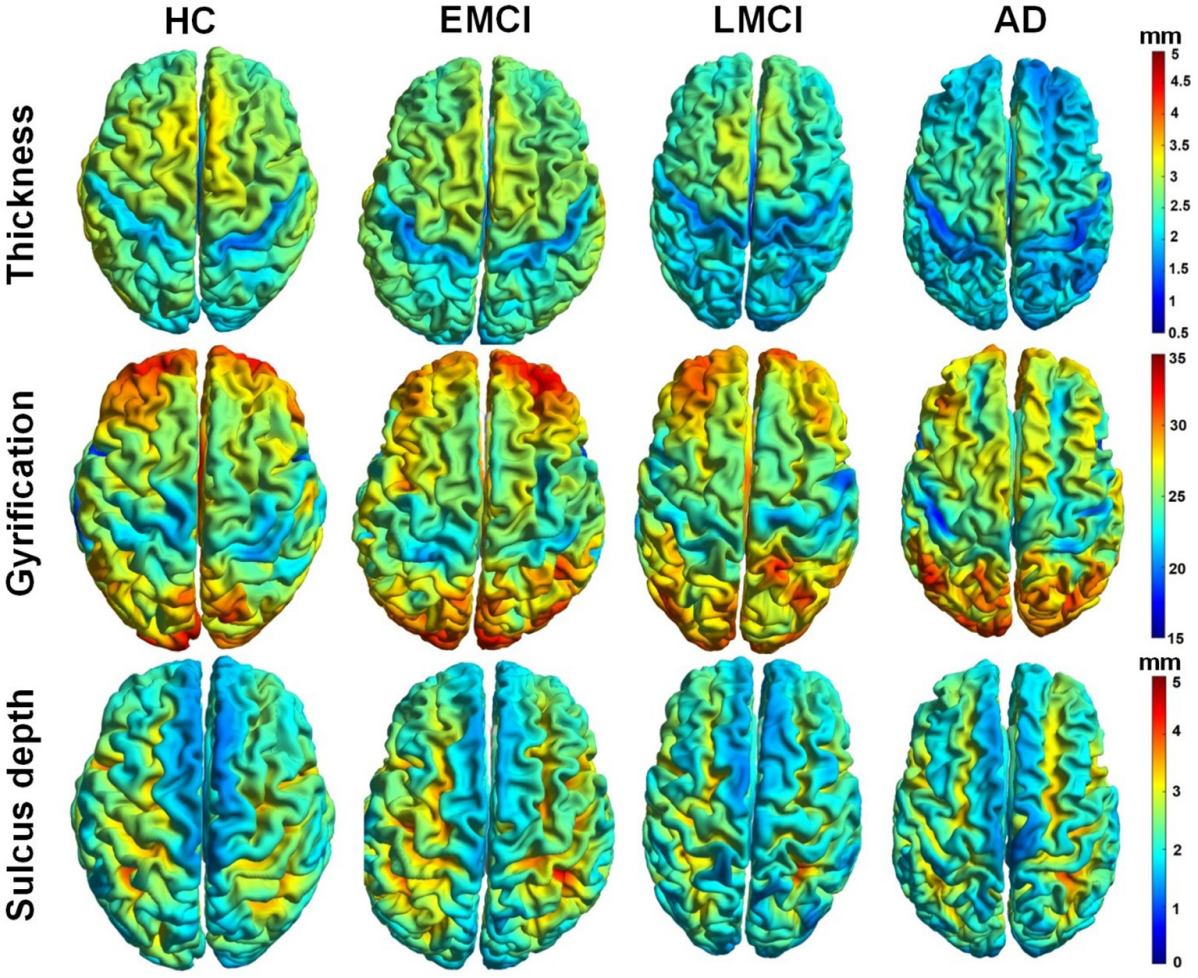
Whole-brain mapping of surface thickness, gyrification index (GI), and sulcus depth maps estimated using CAT12 toolbox. From left to right, each column represents a subject in control, early mild cognitive impairment (EMCI), late MCI (LMCI), and AD groups, respectively.

Figure 3 demonstrated whole-brain mapping of cortical thickness, the GI, and the sulcus depth, where, from left to right, each column represents a subject in HC, EMCI, LMCI, and AD groups. Overall, across the four groups, the distributions of these parameters exhibited similarities. The greatest local cortical thickness appeared to be located in the left and right parietal lobes. The highest local gyrification was located in the frontal lobe, as well as in the occipital lobe, while the lowest GI in the left and right hemispheres appears surrounding the superior parietal gyrus and expanding into the inferior temporal gyrus. The least sulcus depth was detected in the elongated regions along the longitudinal fissure between the left and right hemispheres. As demonstrated in Figure 3, there were some differences in these surface complexity parameters across these groups, especially in cortical thickness (first row in Figure 3). Subsequently, the Kruskal–Wallis and Spearman's correlation tests were used to assess regional differences in these surface morphometry parameters across the groups.

**Figure 4:**
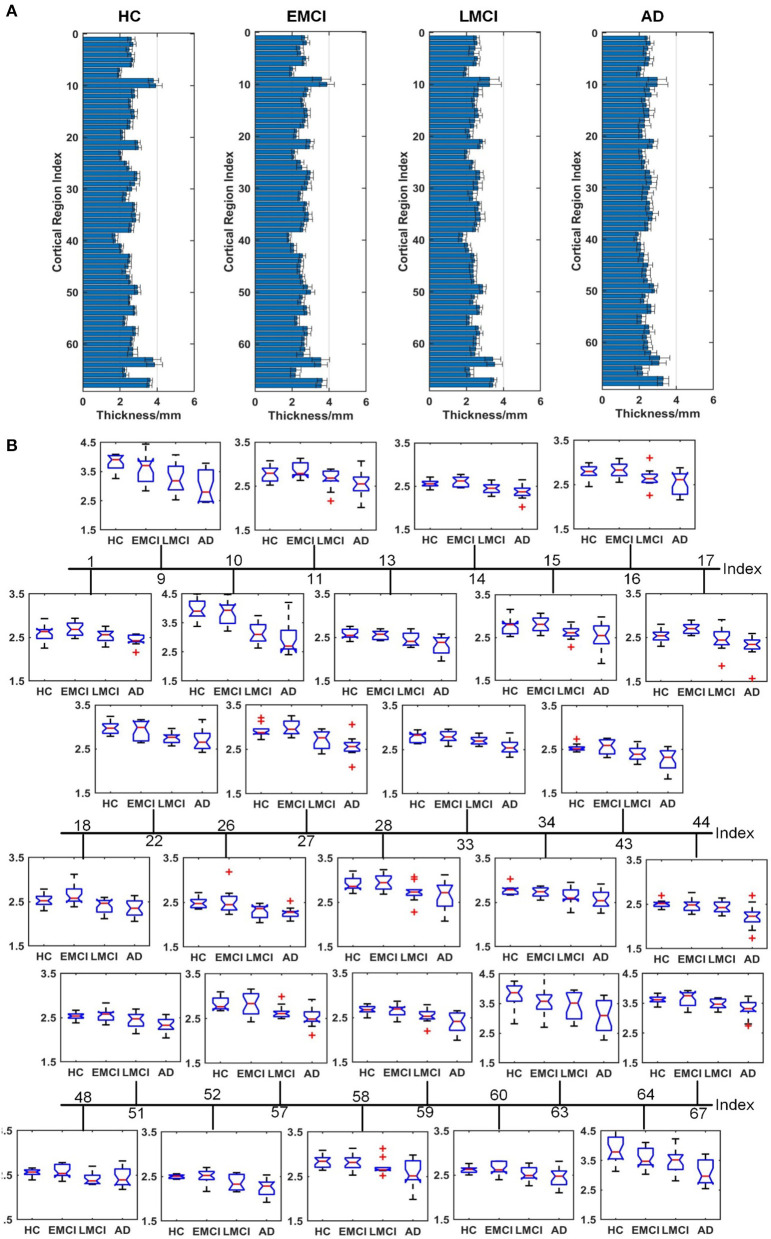
Comparison of cortical thickness across HC, EMCI, LMCI, and AD groups. **(A)** The nodal distribution (mean ± SD) of cortical thickness for each group. **(B)** The Kruskal–Wallis test was performed, and ROIs that exhibit significant difference across four groups were listed. The value of *p* of the Kruskal–Wallis test is reported in Table 3. Region indexes refer to Table 2. Red crosses denote outliers.

**Figure 5:**
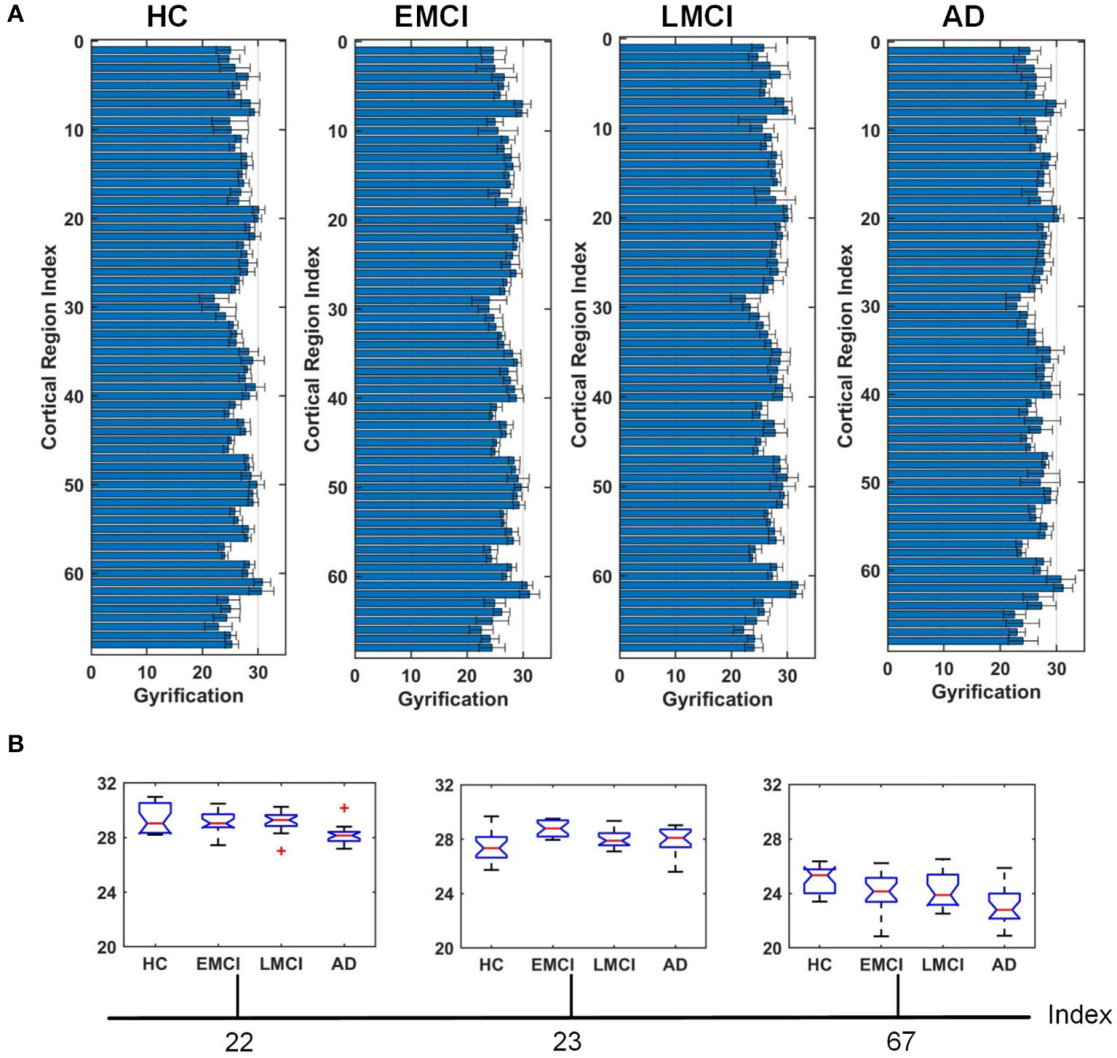
Comparison of the GI across HC, EMCI, LMCI, and AD groups. **(A)** The nodal distribution (mean ± SD) of the GI for each group. **(B)** The Kruskal–Wallis test was performed, and ROIs that exhibit significant difference across four groups were listed. The value of *p* of the Kruskal–Wallis test is reported in Table 3. Region indexes refer to Table 2. Red crosses denote outliers.

**Figure 6:**
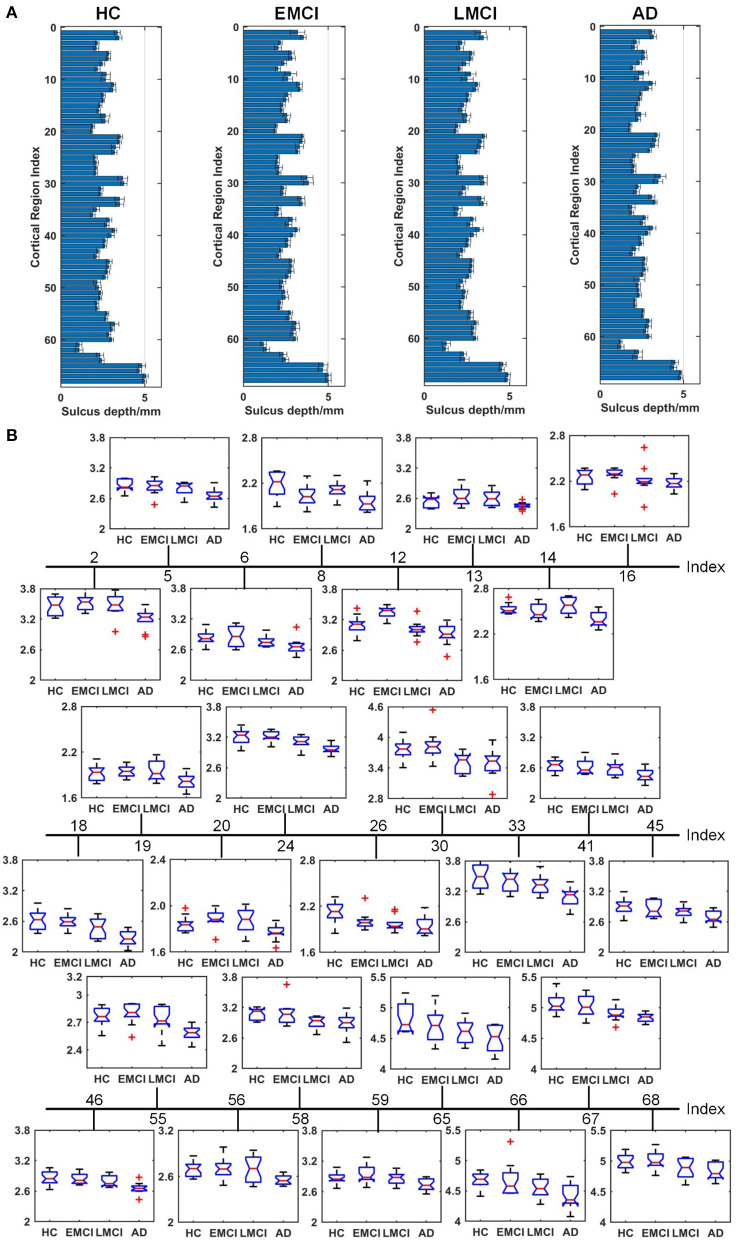
Comparison of the sulcus depth across HC, EMCI, LMCI, and AD groups. **(A)** The nodal distribution (mean ± SD) of the sulcus depth for each group. **(B)** The Kruskal–Wallis test was performed, and ROIs that exhibit significant difference across four groups were listed. The value of *p* of the Kruskal–Wallis test is reported in Table 3. Region indexes refer to Table 2. Red crosses denote outliers.

Figures 4–6 show the nodal distribution (mean ± SD) of cortical thickness, GI, and sulcus depth for each group. After the ROI-based surface complexity was estimated according to the DKT40 atlas, the Kruskal–Wallis test was repeated for 68 regions and the regions that could be significantly identified across four groups were provided in Table 3. As reported in this table, statistically significant differences (*p* < 0.001, uncorrected) in the regions, namely temporal lobe: 1, 9, 10, 11, 15, 16, 27, 28, 57, 58, 63, 64, and 67; frontal lobe: 22, 26, 33, 34, 51, and 52; parietal lobe: 13, 14, 48, 59, and 60; and cingulate: 17, 18, 43, 44, were found in cortical thickness. A significant difference in the GI (*p* < 0.001, uncorrected) was found only in three regions, namely 22, 23, and 67. Significant sulcus depth reductions (*p* < 0.001, uncorrected) over AD progression were revealed, which mainly occurred in the local regions, namely temporal lobe: 2, 12, 16, 58, 65, 66, 67, and 68; frontal lobe: 5, 6, 26, and 33; parietal lobe: 13, 14, 41, 45, 46, 55, 56, and 59; occipital lobe: 8, 19, 20, and 24; and cingulate: 18 and 30.

**Table 3:** The Kruskal–Wallis test on cortical thickness, GI, and sulcus depth across healthy controls (HC), early mild cognitive impairment (EMCI), late MCI (LMCI), and AD groups.

	**Region index**
Cortical thickness	1, 9, 10, 11, 13, 14, 15, 16, 17, 18, 22, 26, 27, 28, 33, 34, 43, 44, 48, 51, 52, 57, 58, 59, 60, 63, 64, 67
Gyrification	22, 23, 67
Sulcus depth	2, 5, 6, 8, 12, 13, 14, 16, 18, 19, 20, 24, 26, 30, 33, 41, 45, 46, 55, 56, 58, 59, 65, 66, 67, 68

*The regions associated with p < 0.001 (uncorrected) were provided*.

**Figure 7:**
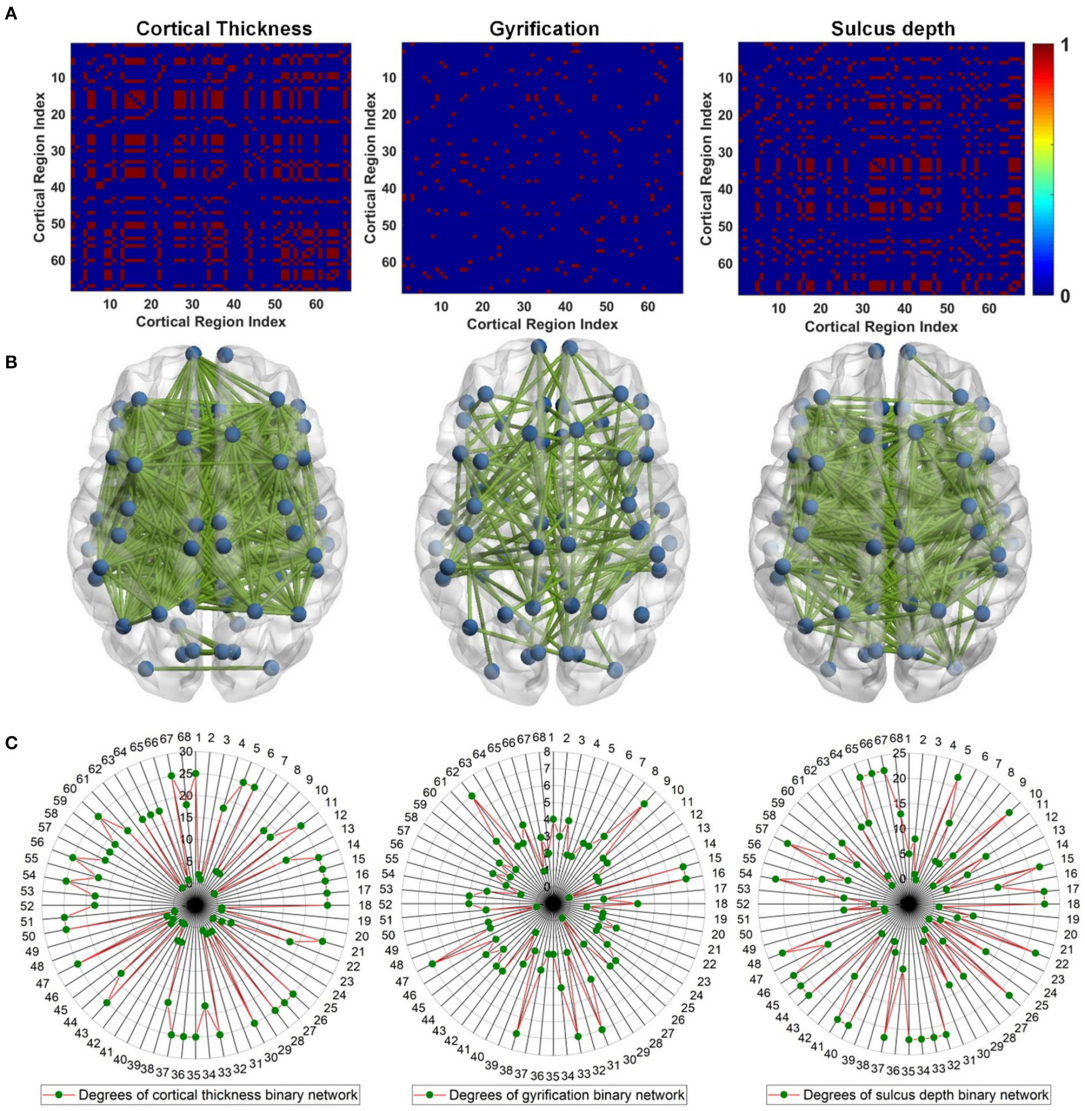
Surface morphometric co-alteration patterns among different cortical subregions. For cortical subregion indices, please refer to Table 2. **(A)** Binarized Spearman's correlation matrices estimated from the ROI-based surface morphometric metrics, including cortical thickness, GI, and sulcus depth. The correlation matrices were thresholded at the value of 1. The value of 1 indicates that the related subregions share the same decreased trend in surface morphometry **(B)** Binary networks correspond to the Spearman's correlation matrices in **(A)**. **(C)** Degree of each cortical subregion estimated from **(A)**.

To reveal gray matter changes occurring simultaneously in different gray matter subregions, we characterized co-alteration patterns of ROI-based surface morphometry during the evolution of AD with the Spearman's correlation analysis. Given the nodes previously designed according to the DKT40 atlas, the co-alteration matrices were constructed for cortical thickness, GI, and sulcus depth. Figure 7A shows the Spearman's correlation matrices between 68 local regions in terms of cortical thickness, GI, and sulcus depth. The matrices were binarized, and the value of +1 indicates a perfect positive correlation, i.e., the related subregions share the same decreased trend in the surface morphometric metrics. The corresponding binary networks were also illustrated in Figure 7B. Interestingly, the three co-alteration networks are different. The node degree is the number of connections that the node has with the other nodes, and it was computed to evaluate co-alteration patterns of the surface morphometric metrics over AD progression (Figure 7C). In the co-alteration network of cortical thickness, we can find that the degrees of 26 nodes (see Figure 7C) are 25. In the sulcus depth network, the degrees of 23 nodes (see Figure 7C) are >20. However, for the gyrification network, the node degrees are much smaller (see Figure 7C). In accordance with the Kruskal–Wallis test, the metrics of cortical thickness and the sulcus depth are more sensitive and specific in distinguishing MCIs and ADs from HCs.

## Discussion

Alzheimer's disease is a progressive neurodegenerative disease characterized by a decline in memory processing and cognitive function. Gray matter atrophy is considered as a close biological substrate of decline in cognitive functioning (Jack et al., 2010b). In this study, with ROI-based surface morphometry analyses based on brain surface meshes, gray matter alterations over AD progression were investigated. Besides cortical thickness, surface complexity (GI and sulcus depth) was estimated at a local scale, revealing a global reduction in the sulcus depth of the MCI and AD groups. The main findings of this study provide a novel perspective for understanding the pathophysiological mechanisms underlying AD and could potentially enhance the accuracy in the early detection and intervention of AD.

A clear deterioration pattern of gray matter over AD progression was shown with the Kruskal–Wallis test. Cortical thickness and sulcus depth were more pronounced during AD progression (Figures 4, 6), and the GI was found to be significantly different only in three local regions (22, 23, 67) (Figure 5). This finding is broadly consistent with the findings in previous studies. Patients with AD mainly exhibited significant gray matter volume reductions in the hippocampus, the temporal lobes, the precuneus, the cingulate gyrus, the insula, and the inferior frontal cortex (Guo et al., 2010; Moller et al., 2013; Lee et al., 2016; Dicks et al., 2019). Our findings also confirmed that the brain regions exhibiting high topological centrality, considered as brain hubs, are more likely to be affected by AD processes, as they are located at the center of important functional networks (Cauda et al., 2018). As reported in Table 3, areas showing significant statistical decreases include insulae (67 and 68); cingulate cortices (17, 18, 43, and 44); inferior, superior, and middle temporal gyri (15, 16, 27, 28, 57, and 58); middle and inferior frontal (22, 26, 33, 34, 51, and 52); pre- and postcentral gyri (13, 14, 41, 45, 46, 55, and 56). Disruption in these hub regions could impede communication between distinct gray matter regions, resulting in impaired cognitive functioning and the rapid development of AD abnormalities from MCI.

Evidence suggests that pathological alteration occurs long before the onset of clinical AD symptoms due to the toxic effects of amyloid-beta plaques (Chetelat et al., 2010; Johnson et al., 2014; Juan et al., 2015). In previous studies, cortical thickness changes were found to be circumscribed to the left hemisphere in patients with MCI and patients with AD using either VBM (Chetelat et al., 2002; Karas et al., 2003; Thompson et al., 2003) or the surface-based cortical thickness analysis (Lerch et al., 2005; Vivek et al., 2006). Specifically, longitudinal studies showed that the left gray matter loss of medial temporoparietal regions was strongly correlated with worse cognitive performance and that faster leftward reduction of gray matter loss was uncovered in patients with AD (Thompson et al., 2003). Our results indicated that eight regions in the left temporal lobe (1, 9, 11, 15, 27, 57, 63, and 67) and five regions in the right temporal lobe (10, 16, 28, 58, and 64) displayed a significant reduction in cortical thickness, supporting the hypothesis that AD-related cortical thickness reduction predominantly occurs in the left hemisphere. However, this spatial deterioration distribution was not observed in the parietal lobe (left: 13 and 59; right: 14, 48, and 60), the frontal lobe (left: 33 and 51; right: 22, 26, 34, and 52), and the cingulate region (left: 17 and 43; right: 18 and 44). Additionally, in the statistical analysis of sulcus depth, we found that five regions in the left parietal lobe (13, 41, 45, 55, and 59) and three regions (14, 46, and 56) in the right parietal lobe exhibited significant reduction. As the spatial deterioration patterns of MCI and AD may be individually different, a larger sample is needed for testing in the next step.

The pathological brains of patients with MCI and patients with AD are also characterized by structural co-alterations in the gray matter, which tend to follow identifiable network-like patterns (Cauda et al., 2018). The co-alteration patterns of surface morphometry parameters indicated the synchronization of gray matter deterioration between distinct gray matter parcels. Studies have revealed that gray matter co-alteration patterns of patients with MCI and patients with AD have a less optimal topological organization characterized by increased segregation and decreased integration (Yong et al., 2008; Tijms et al., 2013; Romerogarcia et al., 2016). By considering the cortical co-alteration pattern as a graph and by studying its edge strength (Spearman's correlation) features at the network level, the cross effects between AD-related incipient lesions may be disclosed. In this study, correlated changes in cortical thickness, GI, and sulcus depth were used to assess the correlation strength across the whole brain and to investigate temporal differences in cross-cortical correlations between groups. Our results provide evidence that alterations of gray matter thickness and sulcus depth are network-like distributed (Figure 7B). This co-alteration exhibits a topological structure and includes some pathological regions that have been thought to be important functional hubs of the brain. As shown in Figure 7C, the degree of these local regions (Table 3) estimated from the cortical thickness correlation matrix was between 18 and 25, except region 48 (precuneus_right), and the degree extracted from the sulcus depth correlation coefficient was between 8 and 22, except 8 (cuneus_right), 14 (inferiorparietal_right), and 19 (lateraloccipital_left). The finding confirms that the primary deterioration in some atrophic regions might lead to a secondary deterioration in other connected areas. The co-alteration patterns of brain atrophy caused by AD appeared to considerably resemble the patterns of brain structural connections (Cauda et al., 2018). However, from the gray matter co-alteration analyses, we still cannot identify the causal relationship between the altered gray matter parcels. It may be a chance to reveal neuropathological co-alterations patterns in patients with MCI and patients with AD, with a combination of functional and anatomic connectivity estimation.

A novel aspect of this study is the assessment of the ROI-based surface morphometric alteration across HC, EMCI, LMCI, and AD groups. The findings are basically in line with the literature showing the associations of gray matter volume morphometry with MCIs and ADs. This might suggest a greater sensitivity of surface estimates in detecting MCI- and AD-related neurodegeneration compared with gray matter voxel-based morphometry. However, the results in this study have several limitations to be interpreted with caution. First, this study was limited by a relatively small sample size. Although we were able to detect effects with this sample size, a larger sample would be optimal for surface morphometry analysis. Second, there is an increased risk for false-positive results because we used uncorrected (*p* < 0.001) thresholds for surface morphometry analysis due to our sample size. Third, brain parcellation may influence the characterization of surface morphometry during AD progression (Messe, 2019; Wu et al., 2019), which deserves further study. Last, the education information of participants and neuropsychological markers are not available in the ADNI database, so they have not been taken into account in the statistical analysis in this study. Despite these limitations, to our knowledge, this is the first report to show the association of brain regional gray matter surface complexity with AD progression. Further, multimodal neuroimaging studies are needed to investigate associations between regional structural brain atrophy and cognition declines in patients with AD. More rigorous methods to combine multimodal MRI brain imaging (structural MRI, diffusion MRI, and functional MRI) may be required. Combining structural brain imaging and connectivity for *in vivo* tracking of AD-related lesions in the asymptomatic stages may be a promising method, facilitating an understanding of how the co-alteration patterns found in this study were constrained by structural or functional connectivity.

## Conclusion

This study reported the ROI-based surface morphometry of gray matter across HC, EMCI, LMCI, and AD groups and identified characteristic alteration patterns in surface morphometry during AD progression. Patients with MCI and patients with AD showed considerable reduction in cortical thickness and surface complexity indices. These parameters could potentially serve as biomarkers for the prediction of AD progression. Future longitudinal studies should determine whether these markers are able to detect gray matter changes with therapies aimed at slowing the disease progression. The possibility of combining structural brain imaging and anatomical or functional connectivity for *in vivo* tracking of AD-linked lesions in the asymptomatic stages is worth further exploration.

## Data Availability Statement

Publicly available datasets were analyzed in this study. This data can be found at: http://adni.loni.usc.edu/.

## Ethics Statement

Ethical review and approval was not required for the study on human participants in accordance with the local legislation and institutional requirements. Written informed consent for participation was not required for this study in accordance with the national legislation and the institutional requirements.

## Author Contributions

ZW: data analysis, result interpretation, manuscript drafting and revision. YP: result interpretation, manuscript drafting, and revision. MH: data analysis and manuscript drafting. YZ: data analysis, result interpretation, and manuscript revision. All authors contributed to the article and approved the submitted version.

## Conflict of Interest

The authors declare that the research was conducted in the absence of any commercial or financial relationships that could be construed as a potential conflict of interest.
